# The association between telomere length and non-alcoholic fatty liver disease: a prospective study

**DOI:** 10.1186/s12916-023-03136-7

**Published:** 2023-11-09

**Authors:** Linxi Tang, Dankang Li, Yudiyang Ma, Feipeng Cui, Jianing Wang, Yaohua Tian

**Affiliations:** 1 Ministry of Education Key Laboratory of Environment and Health, and State Key Laboratory of Environmental Health (Incubating), School of Public Health, Tongji Medical College, Huazhong University of Science and Technology, No.13 Hangkong Road, Wuhan, 430030 China; 2 Department of Maternal and Child Health, School of Public Health, Tongji Medical College, Huazhong University of Science and Technology, No.13 Hangkong Road, Wuhan, 430030 China; 3 School of Population Medicine and Public Health, Chinese Academy of Medical Sciences/Peking Union Medical College, No.31, Beijige-3, Dongcheng District, Beijing, 100730 China

**Keywords:** Telomere length, Genetic susceptibility, Air pollution, Lifestyle, Non-alcoholic fatty liver disease

## Abstract

**Background:**

Research on the association between telomere length (TL) and incident non-alcoholic fatty liver disease (NAFLD) is limited. This study examined this association and further assessed how TL contributes to the association of NAFLD with its known risk factors.

**Methods:**

Quantitative PCR (polymerase chain reaction) was employed to assess leucocyte telomere length. Polygenic risk score (PRS) for NAFLD, air pollution score, and lifestyle index were constructed. Cox proportional hazard models were conducted to estimate the hazard ratios (HRs) and 95% confidence intervals.

**Results:**

Among 467,848 participants in UK Biobank, we identified 4809 NAFLD cases over a median follow-up of 12.83 years. We found that long TL was associated with decreased risk of incident NAFLD, as each interquartile range increase in TL resulted in an HR of 0.93 (95% CI 0.89, 0.96). TL partly mediated the association between age and NAFLD (proportion mediated: 15.52%). When assessing the joint effects of TL and other risk factors, the highest risk of NAFLD was found in participants with low TL and old age, low TL and high air pollution score, low TL and unfavorable lifestyle, and low TL and high PRS, compared to each reference group. A positive addictive interaction was observed between high PRS and low TL, accounting for 14.57% (2.51%, 27.14%) of the risk of NAFLD in participants with low telomere length and high genetic susceptibility.

**Conclusions:**

Long telomere length was associated with decreased risk of NAFLD incidence. Telomere length played an important role in NAFLD.

**Graphical Abstract:**

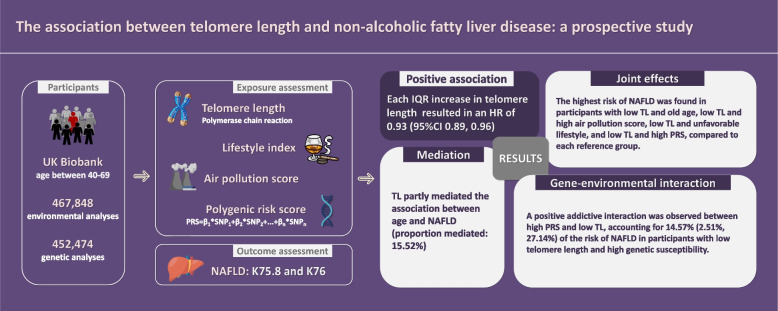

**Supplementary Information:**

The online version contains supplementary material available at 10.1186/s12916-023-03136-7.

## Background

Non-alcoholic fatty liver disease (NAFLD), along with its progressive subtype non-alcoholic steatohepatitis (NASH), are chronic metabolic disorders characterized by excessive hepatic steatosis without a history of alcohol abuse or other liver diseases [1, 2]. Currently, NAFLD is the most common chronic liver disease, affecting approximately 29.9–34.1% of adults globally [3]. NAFLD accounts for the majority of liver-related mortality across the globe [4] and has been linked to other chronic diseases, namely diabetes mellitus and hypertension [5]. Uncovering risk factors and pathogenesis of the disease might be advantageous for identifying at-risk individuals and developing effective interventions.

In particular, NAFLD appears more prevalent among the older population [6], indicating a possible linkage between NAFLD and the aging process. Cellular senescence has generally been identified as a significant mechanism of aging-associated dysfunction. Chief among the drivers of cellular senescence is telomere attrition [7]. Telomeres are nucleoprotein complexes attached to the ends of eukaryotic chromosomes [8]. They are fundamental to maintaining genome stability, as with cellular divisions, telomeres shorten, and a critical decrease in telomere length (TL) will initiate cellular senescence [9]. Accordingly, TL is widely acknowledged as a biomarker of senescence.

Previous research has reported evidence on the involvement of telomere homeostasis in NAFLD development. One animal study found that in established models, *tert*-deficient (deficiency of telomerase reverse transcriptase) mice were more susceptible to hepatocyte injury and steatosis when given liquid high-fat diets, indicating that the absence of telomerase, which is essential for maintaining proliferative capacity and alleviating telomere attrition, could provoke hepatocyte metabolic dysfunction [10]. Meanwhile, population-based investigations have looked into the association between TL and NAFLD via case-control and cross-sectional designs [11, 12]. Nevertheless, the results are inconsistent, and the existing literature lacks longitudinal evidence and cannot infer causal or temporal relationships.

Furthermore, accumulating evidence has demonstrated that NAFLD etiology is attributed to a combination of behavioral, environmental, and genetic factors. Specifically, apart from dietary and exercise-related factors [13, 14], air pollution is also considered to contribute to NAFLD incidence [15]. In addition, genome-wide association studies (GWAS) have uncovered susceptibility loci for NAFLD in European ancestry [16]. However, what still needs to be clarified is how TL contributes to the association between the above-mentioned factors and NAFLD.

Therefore, utilizing the longitudinal design and comprehensive data on lifestyle, air pollution and genetic variations in the UK Biobank, we performed an analysis to examine the association between TL and NAFLD incidence and further assessed how TL contributes to the association of NAFLD with its corresponding risk factors (aging, lifestyle, air pollution and genetic susceptibility).

## Methods

### Study population

The UK Biobank is a prospective research project. It enrolled a cohort of more than half a million people aged 40–69 across the country. In brief, during baseline (2006–2010), participants completed collections of biological samples, physical measurements, and touch-screen questionnaires. They also consented to follow-up through record linkage. Research ethics approval has been granted for the study by the North West Multicenter Research Ethical Committee. The detailed procedure is available at https://www.ukbiobank.ac.uk/media/gnkeyh2q/study-rationale.pdf.

### TL assessment

Leukocyte telomere length (LTL) was assessed via quantitative PCR (polymerase chain reaction) technique. TL was quantified as a ratio of telomere repeat copy numbers to single gene copy numbers. Researchers repeated the measurements and verified the coefficient of variation to guarantee the reliability and consistency of the assessment. The indicator was further log_e_-transformed and *z*-standardized, taking into account differences between laboratories in calibration samples and standard curves. The protocols and procedures applied to quality control parameters can be found elsewhere [17].

### PRS calculation

The UK Biobank’s genotyping process and quality control can be found at https://biobank.ndph.ox.ac.uk/showcase/ukb/docs/ukb_dna_processing.pdf. The polygenic risk score (PRS) was computed using the NAFLD-associated single nucleotide polymorphisms (SNPs) identified in a previous GWAS study of European ancestry [16], as shown in Additional file 1: Table S1. Ten SNPs (rs1260326, rs1919127, rs2068834, rs9992651, rs13118664, rs58542926, rs8107974, rs17216588, rs10500212, rs738409) were incorporated to determine NAFLD PRS (minor allele frequency > 0.05). The weighted PRS method was applied, with each SNP encoded as 0, 1, 2 based on the count of risk alleles and weighted with risk estimates (the natural logarithm of odds ratio) obtained from the GWAS analysis. The equation was as follows: PRS = *β*_1_ × SNP_1_ + *β*_2_ × SNP_2_ + … + *β*_n_ × SNP_n_. The PRS exhibited a range spanning from −1.1936 to 6.494, where a higher score indicates a larger genetic susceptibility to NAFLD onset. The PRS was further categorized into tertiles (low, intermediate, and high genetic risk). Additional file 1: Table S2 shows the association between PRS and NAFLD incidence. After full adjustment, participants who had intermediate or high genetic risk were more likely to develop NAFLD (HR_intermediate_: 1.15 95% CI 1.06, 1.24; HR_high_: 1.60 95% CI 1.48, 1.72), in comparison to those at low genetic risk (*P* for trend = 0.0001).

### Outcome ascertainment

According to the Expert Panel Consensus Statement [18], NAFLD (including NASH) was defined by the International Classification of Diseases Tenth Revisions (ICD-10) code K75.8 and K76. We further separated NAFLD and NASH into K76.0 and K75.8 as secondary outcomes. In accordance with previous studies [15, 19], diagnosis of outcomes and date of incident were ascertained by linking hospital inpatient records. Follow-up period was from recruitment registration to NAFLD or censoring. Censoring was determined by the end of available follow-up (January 1, 2022) or the time of death.

Furthermore, 40,532 participants in UK Biobank attended imaging visit in 2014. Data regarding liver proton density fat fraction (PDFF) was obtained from magnetic resonance images (MRI) assessment. According to existing literature concerning the generally recognized risk threshold, PDFF-defined NAFLD was identified as PDFF > 5% [20, 21].

### Covariates

Age, gender, ethnicity, Townsend deprivation index, body mass index (BMI), cholesterol level, history of diabetes, history of hypertension, lifestyle index, and air pollution score were considered potential covariates, based on previous research. Age and gender were self-reported at baseline. Ethnicity was also self-reported and divided into six groups (white, mixed, Asian or Asian British, Black or Black British, Chinese, and other ethnic groups). Computed according to the postcode of residence, the Townsend deprivation index provided a measure of socioeconomic status (information on employment, population density, automobile availability, and homeownership) at the area level [22]. BMI was assessed by dividing weight (kg) by the square of height (m). Cholesterol level was assessed with venous blood samples. History of diabetes and hypertension were reported by participants in the questionnaire of medical condition. Comprehensive reports about the measurements of covariates can be accessed at https://www.ukbiobank.ac.uk/. The methodology for the construction of lifestyle index and air pollution score was summarized in Additional file 1: Text S1-2 [23–29].

### Analytical cohort

**Fig. 1:**
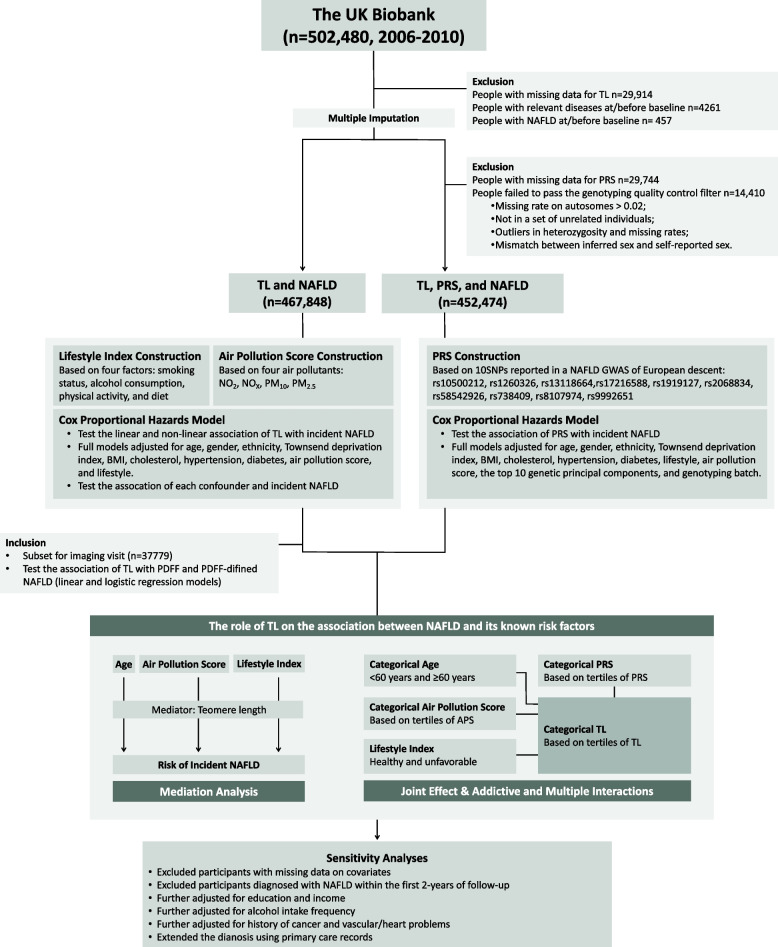
Study design and workflow. Abbreviations: NAFLD, non-alcoholic fatty liver disease; TL, telomere length; CI, confidence interval; PDFF, proton density fat fraction; TDI, Townsend deprivation index; APS, air pollution score; PRS, polygenic risk score; NO_2_, nitrogen dioxide; NO_X_, nitrogen oxides; PM_10_, particulate matter with diameter ≤ 10 μm; PM_2.5_, fine particulate matter with diameter ≤ 2.5 μm

Design and workflow of the study are presented in Fig. 1. Detailed information on exclusion is summarized in Additional file 1: Table S3. In total, 467,848 individuals were included in the primary analysis. With genetic data incorporated, 452,474 individuals were included who passed the genotyping quality control filter with complete required information.

### Statistical analyses

R (4.2.1) and SAS (9.4) were applied to perform the analyses. Statistical significance was determined as a two-sided *P* value of less than 0.05. Participants’ characteristics at baseline were reported as counts (percentages) for categorical variables and means ± standard deviations for continuous variables. A comparison of baseline characteristics by NAFLD was performed by two-sample *t*-test, Fisher’s exact test, or chi-square test. Missing categorical variables were repaired with missing indicators, whereas missing continuous variables were fixed by applying multiple imputations with fully conditional specification (FCS) methods.

To evaluate the association between TL and NAFLD incidence, the hazard ratio (HR) and 95% confidence interval (CI) were assessed using Cox proportional hazard models. Schoenfeld residuals were used to examine the proportional hazards assumption. In addition, the associations of TL with PDFF and PDFF-defined NAFLD were evaluated using linear regression models and logistic regression models. Several confounders were incorporated in the models, namely age, gender, Townsend deprivation index, ethnicity, BMI, diabetes, hypertension, cholesterol levels, lifestyle index, and air pollution score. We additionally included genotyping batch and the top ten genetic principal components for adjustment in the genetic analyses. The exposure-response association of TL with NAFLD incidence was assessed by restricted cubic spline models (RCS).

To assess how TL contributes to the association of NAFLD and its corresponding risk factors, we established causal mediation models to calculate the mediation proportion by TL for the association between NAFLD and age, lifestyle index, and air pollution score using PROC CAUCALMED in SAS v9.4. The mediation analyses consist of three steps to generate the result. First, NAFLD incidence was regressed by age, lifestyle index, and air pollution score, TL and confounders in Cox models. Afterwards, TL (the mediator) were regressed by age, lifestyle index, and air pollution score respectively in linear models adjusting for confounders. Subsequently, the models were combined to measure the natural direct effect (NDE) and natural indirect effect (NIE). NIE/total effect was used to determine the mediation proportion [30, 31]. We assumed that age, gender, ethnicity, Townsend deprivation index, history of diabetes and hypertension, cholesterol, and BMI to be the common set of confounders for exposure-outcome, exposure-mediator, and mediator-outcome associations. In order to guarantee the reliability of mediation analyses, it is imperative to address two key factors: no unmeasured confounding and the model specification. In light of this, the findings need to be interpreted with caution. Then, we assessed the joint effects of TL and age, lifestyle index, air pollution score, and PRS on NAFLD. Furthermore, addictive interaction was evaluated with the relative excess risk due to interaction (RERI) and the attributable proportion due to interaction (AP) [32]. Multiplicative interaction was analyzed by setting cross-product terms in the Cox models. The results were also tested for robustness using four sensitivity analyses, details of which were summarized in Additional file 1: Text S3. All the information in regards to the UK Biobank columns and field ID used in our study was summarized in Additional file 1: Table S4.

## Results

### Baseline characteristics

During a median follow-up of 12.83 years, 4809 NAFLD cases were identified in a total of 467,848 participants in the analytic sample. Table 1 shows the baseline characteristics classified by NAFLD incidence. Additionally, comparing with the UK Biobank full sample demonstrated the representativeness of our analytic population (Additional file 1: Table S5). NAFLD-diagnosed participants were older, with higher Townsend deprivation index, less likely to be white, had higher BMI, history of diabetes and hypertension, and lower cholesterol level. They also tended to have unfavorable lifestyle, higher air pollution levels, and shorter telomere length.

**Table 1:** Baseline characteristics of participants and stratifies by NAFLD status at follow-up

Variables	Total (*N* = 467,848)	Without NAFLD (*N* = 463,039)	With NAFLD (*N* = 4809)	*P* value
Age, years (mean ± SD)	56.54 ± 8.10	56.54 ± 8.10	57.24 ± 7.82	< .0001
60	264,770 (56.6%)	262,152 (56.6%)	2618 (54.4%)	0.003
≥ 60	203,078 (43.4%)	200,887 (43.4%)	2191 (45.6%)	
Gender, *n* (%)				0.069
Female	254,666 (54.4%)	252,111 (54.4%)	2555 (53.1%)	
Male	213,182 (45.6%)	210,928 (45.6%)	2254 (46.9%)	
Ethnicity, *n* (%)				0.001
White ethnicity	423,403 (90.5%)	419,120 (90.5%)	4283 (89.1%)	
Mixed ethnicity	17,114 (3.7%)	16,918 (3.7%)	196 (4.1%)	
Asian ethnicity	16,292 (3.5%)	16,114 (3.5%)	178 (3.7%)	
Black ethnicity	2630 (0.6%)	2595 (0.6%)	35 (0.7%)	
Chinese ethnicity	1431 (0.3%)	1419 (0.3%)	12 (0.2%)	
Other ethnicities	4162 (0.9%)	4096 (0.9%)	66 (1.4%)	
Missing	2816 (0.6%)	2777 (0.6%)	39 (0.8%)	
TDI, (mean ± SD)	− 1.33 ± 3.07	− 1.34 ± 3.06	− 0.36 ± 3.45	< .0001
Missing	572 (0.1%)	567 (0.1%)	5 (0.1%)	
BMI, kg/m^2^ (mean ± SD)	27.42 ± 4.77	27.38 ± 4.74	31.45 ± 5.64	< .0001
Normal weight	154,047 (32.9%)	153,596 (33.2%)	451 (9.4%)	< .0001
Overweight	198,449 (42.4%)	196,739 (42.5%)	1710 (35.6%)	
Obesity	113,500 (24.3%)	110,885 (23.9%)	2615 (54.4%)	
Missing	1852 (0.4%)	1819 (0.4%)	33 (0.7%)	
Cholesterol, mmol/L (mean ± SD)	5.70 ± 1.12	5.70 ± 1.12	5.48 ± 1.23	< .0001
Missing	21,414 (4.6%)	21,151 (4.6%)	263 (5.5%)	
Diabetes, *n* (%)	24,567 (5.3%)	23,693 (5.1%)	874 (18.2%)	< .0001
Hypertension, *n* (%)	112,423 (24.0%)	110,510 (23.9%)	1913 (39.8%)	< .0001
Missing	12,325 (2.6%)	12,072 (2.6%)	253 (5.3%)	
Lifestyle, *n* (%)				< .0001
Unfavorable	219,009 (46.8%)	216,576 (46.8%)	2433 (50.6%)	
Healthy	214,285 (45.8%)	212,514 (45.9%)	1771 (36.8%)	
Missing	34,554 (7.4%)	33,949 (7.3%)	605 (12.6%)	
Air pollution score (mean ± SD)	53.87 ± 8.18	53.85 ± 8.17	55.41 ± 8.49	< .0001
Missing	38,436 (8.2%)	38,229 (8.3%)	207 (4.3%)	
Adjusted T/S ratio (mean ± SD)	0.83 ± 0.13	0.83 ± 0.13	0.82 ± 0.13	< .0001

*Abbreviations*: *NAFLD* Non-alcoholic fatty liver disease, *SD* Standard deviation, *TDI* Townsend deprivation index, *BMI* Body mass index

Continues variables displayed as means ± SD and categorical variables are displayed as numbers (percentages)

### Association between TL and NAFLD incidence

**Fig. 2:**
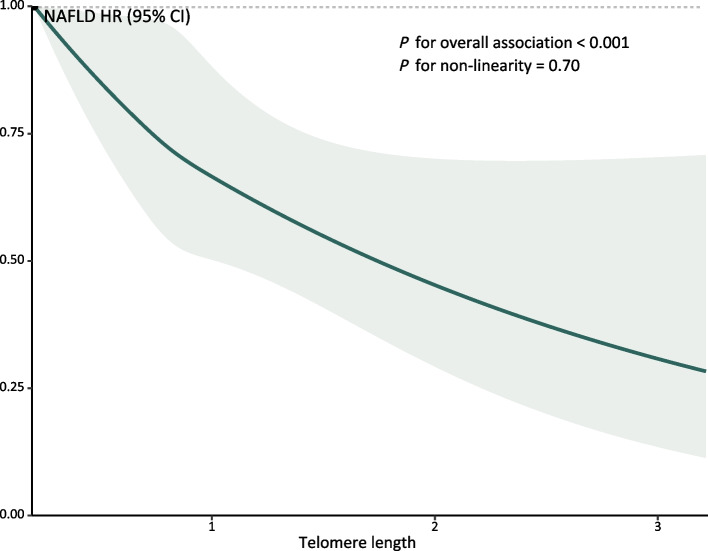
Exposure-response relationships of telomere length and the risk of NAFLD. Abbreviations: NAFLD, non-alcoholic fatty liver disease; HR, hazards ratio; CI, confidence interval. Adjusted for age, gender, ethnicity, Townsend deprivation index, BMI, cholesterol, hypertension, diabetes, lifestyle, and air pollution score

Table 2 presents the association between TL and NAFLD incidence. After full adjustment, the HR for the highest quartile of TL associated with NAFLD incidence was 0.87 (95% CI 0.81, 0.95), compared with the lowest quartile. Each interquartile range (IQR) increase in TL resulted in an HR of 0.93 (95% CI 0.89, 0.96). The RCS models displayed a monotonic exposure-response association of TL with NAFLD risk (*P* for non-linear association = 0.70, Fig. 2). The individual associations for each covariate are summarized in Additional file 1: Table S6. Results of the sensitivity analysis did not differ significantly from those of the current analysis (Additional file 1: Tables S7–S12).

**Table 2:** Association between telomere length and NAFLD incidence

Variables	Cases	*N*	HR (95% CI)		
			Model 1	Model 2	Model 3
NAFLD (K75.8, K76.0)					
Continuous, per IQR increase	4809	467,848	0.88 (0.84, 0.91)	0.92 (0.89, 0.96)	0.93 (0.89,0.96)
Q1	1356	116,961	Ref	Ref	Ref
Q2	1252	116,963	0.91 (0.84, 0.99)	0.95 (0.88, 1.02)	0.95 (0.88,1.03)
Q3	1128	116,963	0.82 (0.76, 0.89)	0.88 (0.81, 0.96)	0.89 (0.82,0.96)
Q4	1073	116,961	0.77 (0.72, 0.84)	0.87 (0.80, 0.94)	0.87 (0.81,0.95)
*P* for trend			< .0001	0.0001	0.0003
NAFLD (K76.0)					
Continuous, per IQR increase	4570	467,848	0.87 (0.84,0.91)	0.92 (0.88,0.95)	0.92 (0.89,0.96)
Q1	1287	116,961	Ref	Ref	Ref
Q2	1190	116,963	0.91 (0.84,0.99)	0.95 (0.87,1.02)	0.95 (0.88,1.03)
Q3	1076	116,963	0.82 (0.76,0.89)	0.88 (0.81,0.96)	0.88 (0.81,0.96)
Q4	1017	116,961	0.77 (0.71,0.84)	0.86 (0.79,0.93)	0.86 (0.80,0.94)
*P* for trend			< .0001	< .0001	0.0002
NASH (K75.8)					
Continuous, per IQR increase	465	467,848	0.83 (0.74,0.94)	0.94 (0.83,1.06)	0.94 (0.84,1.06)
Q1	135	116,961	Ref	Ref	Ref
Q2	133	116,963	0.97 (0.77,1.24)	1.06 (0.83,1.35)	1.06 (0.84,1.35)
Q3	102	116,963	0.74 (0.58,0.96)	0.87 (0.67,1.13)	0.88 (0.68,1.14)
Q4	95	116,961	0.69 (0.53,0.90)	0.88 (0.67,1.15)	0.88 (0.68,1.16)
*P* for trend			0.001	0.17	0.20

*P* value for trend calculated treating the telomere length concentrations (quartile) as a continuous variable

Model 1: Unadjusted

Model 2: Adjusted for age, gender, ethnicity, Townsend deprivation index, BMI, cholesterol, hypertension, and diabetes

Model 3: Model 2 + lifestyle and air pollution score

*Abbreviations*: *NAFLD* Non-alcoholic fatty liver disease, *NASH* Non-alcoholic steatohepatitis, *HR* Hazards ratio, *CI* Confidence interval, *IQR* Interquartile range, *Ref*. Reference

Additional file 1: Table S13 presents the associations of TL with PDFF and PDFF-defined NAFLD. In the subset of imaging visit (*n* = 37,779), 9136 were identified to have NAFLD (PDFF > 5.5%). After full adjustment, we found that every IQR increase in TL associated with −0.11 (95% CI −0.17, −0.05) change of PDFF as well as 0.95 (95% CI 0.92,0.98) of OR for PDFF-defined NAFLD.

### The role of TL on the association of NAFLD with its known risk factors

**Fig. 3:**
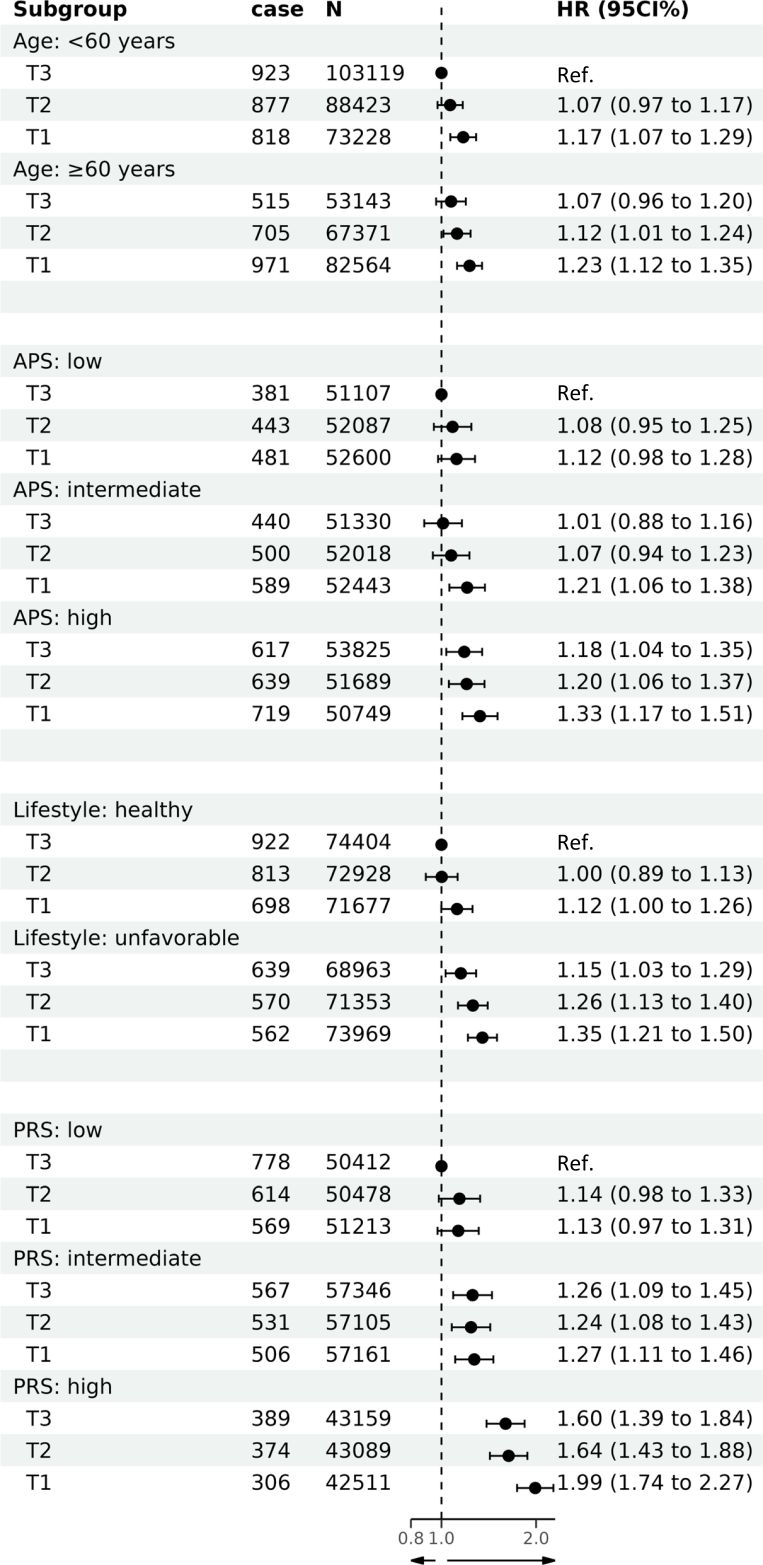
Joint effects of telomere length and known risk factors on NAFLD. Abbreviations: NAFLD, non-alcoholic fatty liver disease; HR, hazards ratio; CI, confidence interval; TDI, Townsend deprivation index; APS, air pollution score; PRS, polygenic risk score; T1, T2, and T3, first, second and third tertile of telomere length. For each analysis, the first groups were the reference categories, as the HR (95% CI) was 1.00 (1.00 to 1.00). Genetic analysis adjusted for age, gender, ethnicity, Townsend deprivation index, BMI, cholesterol, hypertension, diabetes, lifestyle, air pollution score, the top 10 genetic principal components, and genotyping batch. Other analysis adjusted for age, gender, ethnicity, Townsend deprivation index, BMI, cholesterol, hypertension, diabetes, lifestyle and air pollution score

Table 3 displays the associations of NAFLD with age, air pollution score, and lifestyle index mediated by TL. The results indicated that the association of age and NAFLD incidence was partly mediated by TL (proportion mediated: 15.52%), while only a small proportion of the risk of NAFLD incidence attributable to air pollution and lifestyle was explained by TL (proportion mediated: −0.4% and 1.58%, respectively). Figure 3 illustrates the joint effects of TL and other risk factors on NAFLD incidence. The highest risk of NAFLD incidence was found among individuals who had low TL and old age, low TL and high air pollution score, low TL and unfavorable lifestyle, and low TL and high PRS, compared to each reference group. Additional file 1: Table S14 presents the additive and multiplicative interactions between telomere length and known risk factors on NAFLD incidence. A positive addictive interaction was observed between high PRS and low TL (RERI: 0.29, 95% CI 0.05, 0.54), indicating a relative excess risk of 0.29, which accounted for 14.57% (2.51%, 27.14%) of the risk of NAFLD incidence in participants with low telomere length and high genetic susceptibility.

**Table 3:** Mediation analysis of the association between NAFLD and known risk factors, with TL as the mediator

	NAFLD HR	95% CI		*P*
Age				
Total effect	1.009	1.006	1.013	< .0001
Natural direct effect	1.008	1.004	1.012	< .0001
Natural indirect effect	1.001	1.001	1.002	< .0001
Proportion mediated	15.52			
Air pollution score				
Total effect	1.008	1.004	1.012	< .0001
Natural direct effect	1.008	1.004	1.012	< .0001
Natural indirect effect	1.000	1.000	1.000	0.029
Proportion mediated	−0.40			
Lifestyle index				
Total effect	1.209	1.133	1.284	< .0001
Natural direct effect	1.205	1.130	1.280	< .0001
Natural indirect effect	1.003	1.001	1.004	0.0003
Proportion mediated	1.58			

Age and air pollution score were used as continuous variables, while lifestyle index was a binary variable (healthy and unfavorable)

*Abbreviations*: *TL* Telomere length, *NAFLD* Non-alcoholic fatty liver disease, *HR* Hazards ratio, *CI* Confidence interval

## Discussion

In this prospective study of over 450,000 individuals in the UK, our findings suggested an inverse association of TL with NAFLD incidence. In addition, the association of age and NAFLD incidence was partly mediated by TL. The highest risk of NAFLD incidence was found in participants with low TL and old age, low TL and high air pollution score, low TL and unfavorable lifestyle, and low TL and high PRS. In addition, we found a positive addictive interaction between high PRS and low TL, accounting for 14.57% (2.51%, 27.14%) of the risk of NAFLD incidence in participants with low telomere length and high genetic susceptibility.

Previously, the results of the association between TL and NAFLD remained inconsistent. For example, a case-control study involving 70 NAFLD patients and 60 controls found that hepatocyte telomeres (hepatocyte telomere mean fluorescent intensity, MFI) tended to be shorter in NAFLD patients (553 vs 1053, *P* < 0.0001) [12]. In contrast, an age-matched case-control study of 240 diabetics demonstrated that NAFLD patients had significantly longer LTLs (6400.2 ± 71.8 base pairs [bp] vs. 6023.7 ± 49.5 bp, *P* < 0.001) [33]. Additionally, several case-control studies have identified the inverse association [34–36], while others found the association not statistically significant [11, 37]. The generalizability of previous epidemiological studies was largely limited due to their study designs, small sample size, and methods used to define NAFLD, leading to biased results. We conducted a prospective study for the first time in this field to evaluate the association between TL and incident NAFLD as well as the first to identify telomere length as a mediator between age and NAFLD using a population-based approach, though we were unable to identify a significant relationship between TL and NASH incidence, potentially due to limited number of cases (*n* = 465). Numerous studies have suggested the link between TL and progressed liver diseases. In addition to cirrhosis formation being more common in telomerase-deficient mice [38], animal studies have also suggested that telomere system was crucial for hepatocyte regeneration [39]. The associations between TL and other chronic liver diseases have also been revealed in the UK Biobank (alcoholic liver disease and liver cirrhosis) [40]. Population-based research has also found that patients with more advanced stages of liver cirrhosis tended to have shorter TL [41]. The evidence mentioned above, combined with our study, provides valuable insights into the understanding of the potential risk effect of telomere shortening on not only the incidence of NAFLD but also the subsequent progression of advanced chronic liver diseases.

The aging process is accompanied by telomere shortening. Telomere shortening triggers cellular senescence [9] and induces steatosis in hepatocytes via p53-p21 and p16-Rb pathways. IL-1b, IL-6, chemokines, and SASP components are secreted by these senescent cells, causing both tissue degeneration and further senescence. Inflammation was also induced by interleukins and TNF secretion, macrophage activation, and lymphocyte senescence, leading to further progression of NAFLD into NASH [12, 42]. Telomere length in leukocytes is highly correlated with those in other tissues [43], thus serving as a valid proxy for hepatocyte telomere length and providing further explanation for the established association between LTL and NAFLD incidence.

When analyzing the joint effects of TL and other risk factors of NAFLD, we found that participants with low TL and old age, low TL and high air pollution score, low TL and unfavorable lifestyle, and low TL and high PRS exhibited the highest risk of NAFLD onset. The joint effects may be a consequence of common mechanisms including oxidative stress, inflammation, and insulin resistance [13–16]. In addition, we also found that high PRS and low TL interact synergistically to lead to the development of NAFLD. One possible mechanism underlying the synergistic interaction is that both factors may increase level of oxidative stress and inflammation, induced by accelerated telomere shortening and activation of genes that promote NAFLD. On the one hand, oxidative stress can result from DNA damage and telomere shortening [9]. On the other hand, genetic variations (HSD17B13 and TM6SF2) linked to NAFLD possess the capacity to modulate steroid levels and impact autophagy and mitochondrial function in the liver, which subsequently influence the expression or functionality of genes associated with oxidative stress, inflammation, or telomere maintenance [44–46]. Overall, these observations could offer insights for identifying those at-risk and individuals who might gain benefits from interventions to reduce air pollution and cultivate healthy lifestyle.

At present, no drug has been approved to treat or prevent NAFLD. Current management of the disease primarily places emphasis on controlling the metabolic condition, with diet and exercise serving as the mainstays of disease prevention and treatment. Existing literature has suggested some effective interventions for telomere extension, such as danazol [47]. Other therapies targeting reactivation of endogenous TERT expression [48] or exogenous delivery [49] have been proposed. Our study provides evidence for the potential of telomere length as a therapeutic target to reduce NAFLD incidence, which has also been proposed by a previous review on this field [50]. However, the benefits of telomere extension should be considered cautiously, since long telomeres have been implicated in the development of multiple cancers [51]. Studies involving animals and populations should be conducted in the future to investigate possible telomere-lengthening therapies that are capable of preventing and treating NAFLD while also safe for the general public’s health.

Strengths of this research included the large sample size, prospective design, reliable outcome measurement, consistency in the sensitivity analysis, and appropriate adjustment for covariates. It is pertinent to note, however, that this study has some limitations. First of all, UK Biobank is not an accurate representation of the UK population [52]. Thus, the estimation of risk is generalizable [53], though summary statistics including NAFLD incidence are unreliable [54]. Second, the ascertainment of NAFLD cases was based on hospital admission records, thus tended to identify more advanced cases, potentially resulting in the underdiagnosis of milder cases. This is a commonly used strategy for NAFLD identification in previous studies [15, 19, 55, 56], and advanced cases have been proven to be more clinically significant, as the severity was positively correlated with subsequent adverse outcomes [57]. Nevertheless, to address this issue, we extended the diagnosis by using primary care records, and the results remained consistent. In addition, we assessed the association between TL and MRI-derived liver PDFF, as a means of detecting moderate NAFLD cases, and found consistent results, suggesting that TL could not only contribute to advanced cases of NAFLD but also less advanced cases. Third, we acknowledge the proposed new terminology in replacement for NAFLD as metabolic dysfunction-associated steatotic liver disease (MASLD) published in June 2023 [58]. The new definition incorporates the inclusion of metabolic syndrome as a criterion, while the old definition solely relies on histology and ultrasound examination. Nevertheless, considering a body of evidence demonstrating comparable prevalence and the fact that almost all patients with NAFLD meet MASLD criteria [59–62], our findings of the association between TL and NAFLD incidence could be largely extrapolated under the new MASLD definition framework. Still, future studies should be conducted to directly assess the role of TL on MASLD pathogenesis and verify our study. Fourth, air pollution may be underestimated or overestimated since only four air pollutants were measured at participants’ home addresses during a 1-year period (2010) as an indicator of exposure, which was commonly used in previous environmental epidemiological studies [27, 63]. Fifth, lifestyle status was determined using self-reported data, which may result in incorrect classification. Sixth, due to most of participants being of European descent, caution should be exercised when generalizing our results, particularly in relation to genetic susceptibility.

## Conclusions

Long telomere length was associated with decreased risk of NAFLD incidence. The association of age and NAFLD incidence was partly mediated by TL. The highest risk of NAFLD incidence was found in participants with low TL and old age, low TL and high air pollution score, low TL and unfavorable lifestyle, and low TL and high PRS. In addition, we found a positive addictive interaction between high PRS and low TL. Future studies targeting diverse populations should be conducted to evaluate the association of TL and NAFLD pathogenesis and further investigate potential telomere-lengthening therapies that that are capable of preventing and treating NAFLD.

### Supplementary Information

**Additional file 1: Text S1.** Lifestyle index construction. **Text S2.** Air pollution estimates. **Text S3.** Sensitivity analyses. **Table S1.** Summary results of 10 SNPs associated with NAFLD from the study of Quentin et al., 2020. **Table S2.** Association between PRS and NAFLD incidence. **Table S3.** People excluded with relevant diseases at/before baseline. **Table S4.** Information on the UK Biobank columns and field ID used in the current study. **Table S5.** Demographic comparison of study population and UK Biobank full sample. **Table S6.** Associations between telomere length, each confounder and NAFLD. **Table S7.** Sensitivity analysis: after excluding participants with missing data on covariates. **Table S8.** Sensitivity analysis: after excluding participants diagnosed with NAFLD within the first2-years of follow-up. **Table S9.** Sensitivity analysis: further adjusted for education and income. **Table S10.** Sensitivity analysis: further adjusted for alcohol intake frequency. **Table S11.** Sensitivity analysis: further adjusted for history of cancer and vascular/heart problems. **Table S12.** Sensitivity analysis: added data on primary care. **Table S13.** Associations of telomere length with PDFF and PDFF-defined NAFLD. **Table S14.** Additive and multiplicative interactions between telomere length and other risk factors on NAFLD incidence.

## Data Availability

The data used in this current study are available from the UK Biobank data resources. Permissions are required in order to gain access to the UK Biobank data resources, subject to successful registration and application process. Further information can be found on the UK Biobank website (https://www.ukbiobank.ac.uk/).

## Abbreviations

AP: Attributable proportion due to interaction
APS: Air pollution score
BMI: Body mass index
CI: Confidence interval
FCS: Fully conditional specification
GWAS: Genome-wide association studies
HR: Hazard ratio
ICD-10: The International Classification of Diseases Tenth Revisions
IQR: Interquartile range
LTL: Leukocyte telomere length
MASLD: Metabolic dysfunction-associated steatotic liver disease
MRI: Magnetic resonance images
NAFLD: Non-alcoholic fatty liver disease
NASH: Non-alcoholic steatohepatitis
NDE: Natural direct effect
NIE: Natural indirect effect
NO_2_: Nitrogen dioxide
NOX: Nitrogen oxides
PCR: Polymerase chain reaction
PDFF: Proton density fat fraction
PM10: Particulate matter with diameter ≤ 10 μm
PM2.5: Fine particulate matter with diameter ≤ 2.5 μm
PRS: Polygenic risk score
RCS: Restricted cubic spline
Ref.: Reference
RERI: Relative excess risk due to interaction
SD: Standard deviation
SNPs: Single nucleotide polymorphisms
TDI: Townsend deprivation index
TL: Telomere length
