# Supplementary material for: The association between telomere length and non-alcoholic fatty liver disease: a prospective study
Source: BMC Med. 2023 Nov 9;21:427. doi: 10.1186/s12916-023-03136-7 (PMC10634180; doi:10.1186/s12916-023-03136-7)
Supplement: Supplementary file 1 — Additional file 1: Text S1. Lifestyle index construction. Text S2. Air pollution estimates. Text S3. Sensitivity analyses. Table S1. Summary results of 10 SNPs associated with NAFLD from the study of Quentin et al., 2020. Table S2. Association between PRS and NAFLD incidence. Table S3. People excluded with relevant diseases at/before baseline. Table S4. Information on the UK Biobank columns and field ID used in the current study. Table S5. Demographic comparison of study population and UK Biobank full sample. Table S6. Associations between telomere length, each confounder and NAFLD. Table S7. Sensitivity analysis: after excluding participants with missing data on covariates. Table S8. Sensitivity analysis: after excluding participants diagnosed with NAFLD within the first2-years of follow-up. Table S9. Sensitivity analysis: further adjusted for education and income. Table S10. Sensitivity analysis: further adjusted for alcohol intake frequency. Table S11. Sensitivity analysis: further adjusted for history of cancer and vascular/heart problems. Table S12. Sensitivity analysis: added data on primary care. Table S13. Associations of telomere length with PDFF and PDFF-defined NAFLD. Table S14. Additive and multiplicative interactions between telomere length and other risk factors on NAFLD incidence. [file 12916_2023_3136_MOESM1_ESM.docx]

**The association between telomere length and non-alcoholic fatty liver disease: a prospective study**

**Supplementary Materials**

Text S1 Lifestyle index construction

Text S2 Air pollution estimates

Text S3 Sensitivity analyses

Table S1 Summary results of 10 SNPs associated with NAFLD from the study of Quentin. et al., 2020

Table S2 Association between PRS and NAFLD incidence

Table S3 People excluded with relevant diseases at/before baseline

Table S4 Information on the UK Biobank columns and field ID used in the current study

Table S5 Demographic comparison of study population and UK Biobank full sample

Table S6 Associations between telomere length, each confounder and NAFLD

Table S7 Sensitivity analysis: after excluding participants with missing data on covariates

Table S8 Sensitivity analysis: after excluding participants diagnosed with NAFLD within the first2-years of follow-up

Table S9 Sensitivity analysis: further adjusted for education and income

Table S10 Sensitivity analysis: further adjusted for alcohol intake frequency

Table S11 Sensitivity analysis: further adjusted for history of cancer and vascular/heart problems

Table S12 Sensitivity analysis: added data on primary care

Table S13 Associations of telomere length with PDFF and PDFF-defined NAFLD

Table S14 Additive and multiplicative interactions between telomere length and other risk factors on NAFLD incidence

Supplementary reference

**Text S1 Lifestyle index construction**

Four modifiable lifestyle factors were used to calculate the lifestyle index, including smoking status, alcohol consumption status, diet, and physical activity. These factors were determined by participants answering the touchscreen questionnaire at baseline. Smoking status and alcohol consumption were defined as current or not current smokers/drinkers. According to the recommendations of the American Heart Association, regular physical activity was defined as sustaining moderate activity for ≥ 150 min/week or vigorous activity ≥ 75 min/week (or an equivalent combination) or exercising moderately ≥ 5 days/week or vigorously ≥ 1 day/week[23]. A healthy diet was established utilizing seven dietary components (fruit, vegetables, fish, processed meat, unprocessed red meat, whole grain, and refined grain) based on dietary recommendations for cardiometabolic health[24]. The definition of a healthy diet was meeting more than 4 of 7 dietary criteria. Generally, the lifestyle index ranged from 0 to 4, with higher values indicating more adherence to healthy lifestyle practices. We subsequently categorized the lifestyle index into healthy lifestyle (3-4 scores) and unfavorable lifestyle (0-2 scores). A detailed description of the construction criteria and basic statistics of lifestyle index was summarized in **Table A1**.

Table A1 Construction and distribution of lifestyle in the current study

| Healthy lifestyle factors | Definition | Score | N (%) |
| --- | --- | --- | --- |
| Smoking status |  |  |  |
| Current smoker | current smoker | 0 | 48168 (10.3%) |
| Not current smoker | never/former smoking | 1 | 417304 (89.2%) |
| Missing |  |  | 2376 (0.5%) |
| Alcohol consumption |  |  |  |
| Current drinker | current drinker | 0 | 429827 (91.9%) |
| Not current smoker | never/former drinking | 1 | 36904 (7.9%) |
| Missing |  |  | 1117 (0.2%) |
| Physical activity |  |  |  |
| Regular | 1. ≥150 min of moderate activity per week; 2. ≥75 min of vigorous activity per week; 3. ≥150 min of moderate activity and vigorous activity per week 2. ≥5 days of moderate activity per week; 3. ≥1 days of vigorous activity per week | 1 | 117351 (25.1%) |
| Not regular | not up to the standard of regular physical activity | 0 | 325003 (69.5%) |
| Missing |  |  | 25494 (5.4%) |
| Diet |  |  |  |
| Healthy | meeting ≥ 4 of 7 criteria below: 1. Fruits (fresh fruit and dried fruit): ≥3 servings/day; 2. Vegetables (cooked vegetable and salad/raw vegetable): ≥3 servings/day; 3. Fish (oily fish and non-oily fish): ≥2 servings/week; 4. Processed meat: ≤1 servings/week; 5. Unprocessed red meat (beef, lamb/mutton, and pork): ≤1.5 servings/week; 6. whole grains (brown bread, wholemeal and wholegrain bread, bran and oat cereal): ≥3 servings/day; 7. Refined grains (white and other type of bread, biscuit cereal, muesli and other type of cereal): ≤1.5 servings/day. | 1 | 48168 (10.3%) |
| Unfavorable | not up to the standard of healthy diet | 0 | 417304 (89.2%) |
| Missing |  |  | 2376 (0.5%) |
| Lifestyle |  |  |  |
| Healthy | sum score ranged 3-4 | 1 | 48168 (10.3%) |
| Unfavorable | sum score ranged 0-2 | 0 | 417304 (89.2%) |
| Missing |  |  | 2376 (0.5%) |

**Text S2 Air pollution estimates**

In the UK Biobank, the annual average exposure of air pollutants (NO_2_, NO_x_, PM_10_, and PM_2.5_) was estimated using the Land Use Regression (LUR) model from the European Study of Cohorts for Air Pollution Effects (ESCAPE) project[25, 26]. Participants’ residential addresses provided at baseline were linked to geographic information system (GIS) and matched with predictive variables to calculate the air pollutant concentrations (100m*100m resolution) in certain years. According to previous studies, leave-one-out cross-validation presented good model performance for the four air pollutants[27]. A comparison has been made of data on air pollution in the UK Biobank and that in the UK Air Information Resource (<https://uk-air.defra.gov.uk/data/pcm-data>), and the consistency was verified[28]. The average levels of NO_2_, NO_x_, PM_10_, and PM_2.5_ in 2010 were used in this study. The stability of most air pollutants between 2005-2021 has been confirmed by earlier research[27-29] to ensure the representativeness of the chosen data as long-term exposures.

In order to interpret the overall impact of the four air pollutants, we generated a weighted air pollution score. Using the estimated multivariable-adjusted β coefficients of air pollutants on NAFLD (see **Table A2**), we calculated the air pollution score with the following equation: air pollution score = (β[NO_2_] × NO_2_ + β[NO_X_] × NO_X_ + β[PM_10_] × PM_10_ + β[PM_2.5_] × PM_2.5_) ×(β[NO_2_] + β[NO_X_] +β[PM_10_] + β[PM_2.5_]) (2). The air pollution score ranged from 37.75 to 151.24, with a higher score indicating greater exposure to air pollution.

Table A2 Association between air pollutants and NAFLD

| variables | β coefficient | NAFLD adjusted HR | Lower 95% CI | Upper 95% CI |
| --- | --- | --- | --- | --- |
| NO_2_ | 0.01 | 1.01 | 1.00 | 1.01 |
| NO_x_ | 0.003 | 1.00 | 1.00 | 1.01 |
| PM_10_ | 0.01 | 1.01 | 0.99 | 1.03 |
| PM_2.5_ | 0.07 | 1.07 | 1.04 | 1.10 |

Abbreviations: NAFLD, non-alcoholic fatty liver disease; HR, hazards ratio; CI, confidence interval; NO_2_, nitrogen dioxide; NO_X_, nitrogen oxides; PM_10_, particulate matter with diameter ≤10μm; PM_2.5_, fine particulate matter with diameter ≤2.5μm.

Adjusted for age, gender, ethnicity, Townsend deprivation index, BMI, Cholesterol, hypertension, diabetes, and lifestyle.

**Text S3 Sensitivity analyses**

Firstly, we excluded participants with missing covariate data. Secondly, we restricted the analyses among participants diagnosed with NAFLD after the first two years of follow-up to avoid possible reverse causality bias. Thirdly, we further adjusted for education and average total household income in the models to examine whether individual-level socioeconomic status would contribute to the associations studied. Fourthly, we took alcohol intake frequency into account to examine whether alcohol consumption and intake frequency as an independent factor would influence the results. Fifthly, we further adjusted for history of cancer and vascular/heart problem to assess whether these medical conditions would have an impact on the study findings. In accordance with previous studies[8], we extended the diagnosis of NAFLD by using primary care records, available for a limited number of individuals in the UK Biobank, to encompass less severe cases.

Table S1 Summary results of 10 SNPs associated with NAFLD from the study of Quentin. et al., 2020

| SNP | Chromosome | Effect allele | Gene | *P* value | OR(95%CI) |
| --- | --- | --- | --- | --- | --- |
| rs1260326 | 2 | T | *GCKR* | 1.06E-10 | 1.278(1.186-1.377) |
| rs1919127 | 2 | C | *C2orf16* | 5.61E-10 | 1.290(1.190-1.398) |
| rs2068834 | 2 | C | *ZNF512* | 8.49E-11 | 1.302(1.202-1.410) |
| rs9992651 | 4 | A | *HSD17B13* | 2.78E-08 | 0.744(0.671-0.826) |
| rs13118664 | 4 | T | *HSD17B13* | 1.41E-08 | 0.740(0.667-0.821) |
| rs58542926 | 19 | T | *TM6SF2* | 2.05E-11 | 1.609(1.400-1.849) |
| rs8107974 | 19 | T | *SUGP1* | 2.58E-12 | 1.632(1.423-1.872) |
| rs17216588 | 19 | T | *-* | 7.25E-14 | 1.612(1.423-1.827) |
| rs10500212 | 19 | T | *PBX4* | 3.40E-12 | 1.549(1.369-1.752) |
| rs738409 | 22 | G | *PNPLA3* | 1.45E-49 | 1.827(1.687-1.979) |

Table S2 Association between PRS and NAFLD incidence

| Variables | Case | N | NAFLD HR (95%CI) | |
| --- | --- | --- | --- | --- |
|  |  |  | Model 1 | Model 2 |
| continuous | 4634 | 452474 | 1.22 (1.19, 1.26) | 1.23 (1.20, 1.27) |
| Low (- 0.17) | 1069 | 128759 | Ref. | Ref. |
| Intermediate (0.17 - 0.92) | 1604 | 171612 | 1.13 (1.04, 1.22) | 1.15 (1.06, 1.24) |
| High (0.92 - ) | 1961 | 152103 | 1.56 (1.45, 1.68) | 1.60 (1.48, 1.72) |
| *P* for trend |  |  | <.0001 | 0.0001 |

Abbreviations: PRS, polygenic risk score; NAFLD, non-alcoholic fatty liver disease; HR, hazards ratio; CI, confidence interval; Ref, reference.

*P* value for trend calculated treating the PRS concentrations (tertile) as a continuous variable.

Model 1: Unadjusted.

Model 2: Adjusted for age, gender, ethnicity, Townsend deprivation index, BMI, cholesterol, hypertension, diabetes, lifestyle, air pollution score, the top 10 genetic principal components, and genotyping batch.

Table S3 People excluded with relevant diseases at/before baseline

| ICD-10 | Diagnosis |
| --- | --- |
| K70 | Alcoholic liver disease |
| B16,B17,B18, B19 | Viral Hepatitis |
| K83.0, K74.3, K75.4 | Autoimmune liver disease |
| E83.1 | Hemochromatosis |
| E83.0 | Wilson |
| E88.0 | Alpha-1-antirypsin deficiency |
| I82.0, K76.5 | Budd-Chiari |
| K73.9, K73.2 | Chronic hepatitis, unspecified |
| K74.4, K74.5 | Secondary or unspecified biliary cirrhosis |
| F10 | alcohol use disorder |
| F11-F14, F16, F18,F19 | drug use disorders except nicotine and caffeine |

Table S4 Information on the UK Biobank columns and field ID used in the current study

| Categories | UKB column | UKB Field ID |
| --- | --- | --- |
| Exposures | Adjusted T/S ratio | [22191](https://biobank.ndph.ox.ac.uk/showcase/field.cgi?id=22191) |
| Outcomes | Hospital inpatient record | K76.0, K75.8 |
|  | Primary care record | Read_2: J61y1, J61y7, J61y9, J61y8, Jyu72  Read_3: J61y1, J61y7, X307v, XM095, Jyu72, X306p, X306q, XaQIT |
|  | Proton density fat fraction (PDFF) | 40061 |
| Exclusion | Hospital inpatient record | K70, B16, B17, B18, B19, K83.0, K74.3, K75.4, E83.1, E83.0, E88.0, I82.0, K76.5, K73.9, K73.2, K74.4, K74.5, F10, F11-F14, F16, F18, F19 |
|  | Genetic principal components | 22009 |
| PRS | Genomics | rs1260326, rs1919127, rs2068834, rs9992651, rs13118664, rs58542926, rs8107974, rs17216588, rs10500212, rs738409 |
| Air pollution score | Nitrogen dioxide air pollution; 2010  Nitrogen oxides air pollution; 2010  Particulate matter air pollution (pm10); 2010  Particulate matter air pollution (pm2.5); 2010 | 24003, 24004, 24005, 24006 |
| Lifestyle index | Smoking status | 20116 |
|  | Alcohol consumption | 20117 |
|  | Physical activity | 894, 914, 884,904 |
|  | Diet | 1289, 1299, 1309, 1319, 1329, 1339, 1349, 1369, 1379, 1389, 1438, 1448, 1458, 1468 |
| Covariates | Age at recruitment | 21022 |
|  | Sex | 31 |
|  | Ethnic background | 21000 |
|  | Townsend deprivation index | 22189 |
|  | Body mass index (BMI) | 21001 |
|  | Cholesterol | 30690 |
|  | History of diabetes | 2443 |
|  | History of hypertension | 6150 |
| Sensitivity analyses | Education | 6138 |
|  | Average total household income | 738 |
|  | Alcohol intake frequency | 1558 |
|  | History of cancer | 2453 |
|  | History of vascular/heart problem | 6150 |

Table S5 Demographic comparison of study population and UK Biobank full sample

| Variables | Study population (n = 467848) | UKB full sample (n=502480) |
| --- | --- | --- |
| Gender, n (%) |  |  |
| Female | 254666 (54.4%) | 273366 (54.4%) |
| Male | 213182 (45.6%) | 229113 (45.6%) |
| Age, years (mean ± SD) | 56.5 ± 8.1 | 56.5 ± 8.1 |
| Ethnicity, n (%) |  |  |
| White ethnicity | 423403 (90.5%) | 453651 (90.3%) |
| Mixed ethnicity | 17114 (3.7%) | 18862 (3.8%) |
| Asian ethnicity | 16292 (3.5%) | 17521 (3.5%) |
| Black ethnicity | 2630 (0.6%) | 2848 (0.6%) |
| Chinese ethnicity | 1431 (0.3%) | 1574 (0.3%) |
| Other ethnicity | 4162 (0.9%) | 4558 (0.9%) |
| Townsend deprivation index, mean (SD) | -1.3 ± 3.1 | -1.3 ± 3.1 |
| BMI (kg/m^2^), mean (SD) | 27.4 ± 4.8 | 27.4 ± 4.8 |
| Cholesterol, mmol/L (mean ± SD) | 5.7 ± 1.1 | 5.7±1.1 |
| Diabetes, n (%) | 24567 (5.3%) | 26398 (5.28%) |
| Hypertension, n (%) | 112423 (24.0%) | 121190 (24.1%) |
| Lifestyle, n (%) |  |  |
| Unhealthy | 219009 (46.8%) | 233815 (46.5%) |
| Healthy | 214285 (45.8%) | 228574 (45.5%) |
| Air pollution score (mean ± SD) | 53.8 ± 8.4 | 53.9±8.5 |

Abbreviations: SD, standard deviation; BMI, body mass index;

Continues variables displayed as means ± SD, and categorical variables are displayed as numbers (percentages).

Table S6 Associations between telomere length, each confounder and NAFLD

| Variable | HR | lower 95%CI | upper 95%CI |
| --- | --- | --- | --- |
| Telomere length, per IQR increase | 0.93 | 0.89 | 0.96 |
| Age | 1.01 | 1.00 | 1.01 |
| Sex |  | | |
| Female | Ref. | | |
| Male | 0.98 | 0.93 | 1.04 |
| Ethnic |  | | |
| White ethnicity | Ref. | | |
| Mixed ethnicity | 0.92 | 0.79 | 1.06 |
| Asian ethnicity | 1.07 | 0.92 | 1.25 |
| Black ethnicity | 1.17 | 0.84 | 1.63 |
| Chinese ethnicity | 1.23 | 0.70 | 2.17 |
| Other ethnicity | 1.21 | 0.94 | 1.54 |
| TDI | 1.05 | 1.04 | 1.06 |
| BMI | 1.10 | 1.10 | 1.11 |
| Cholesterol | 0.98 | 0.96 | 1.01 |
| Hypertension |  | | |
| No | Ref. | | |
| Yes | 1.40 | 1.32 | 1.50 |
| Diabetes |  | | |
| No | Ref. | | |
| Yes | 2.13 | 1.96 | 2.31 |
| Lifestyle | Ref. | | |
| Unfavorable | Ref. | | |
| Healthy | 0.83 | 0.78 | 0.88 |
| Air pollution score | 1.01 | 1.00 | 1.01 |

Abbreviations: NAFLD, non-alcoholic fatty liver disease; HR, hazards ratio; CI, confidence interval; IQR, interquartile range; Ref, reference; TDI, Townsend deprivation index; BMI, body mass index.

Adjusted for age, gender, ethnicity, Townsend deprivation index, BMI, Cholesterol, high blood pressure, diabetes, lifestyle and air pollution score

Table S7 Sensitivity analysis: after excluding participants with missing data on covariates

| Variables | N | Case | HR | Lower 95%CI | Upper 95%CI |  |
| --- | --- | --- | --- | --- | --- | --- |
|  | Model 1 | | | | | |
| continuous, per IQR increase | 467848 | 4809 | 0.88 | 0.84 | 0.91 |  |
| Q1 | 116954 | 1356 | Ref. | Ref. | Ref. |  |
| Q2 | 116975 | 1252 | 0.91 | 0.84 | 0.98 |  |
| Q3 | 116959 | 1128 | 0.82 | 0.76 | 0.88 |  |
| Q4 | 116960 | 1073 | 0.77 | 0.71 | 0.84 |  |
| *P* for trend |  |  |  |  |  | <.0001 |
|  | Model 2 | | | | | |
| continuous, per IQR increase | 429995 | 4235 | 0.93 | 0.89 | 0.97 |  |
| Q1 | 107229 | 1183 | Ref. | Ref. | Ref. |  |
| Q2 | 107471 | 1103 | 0.96 | 0.89 | 1.04 |  |
| Q3 | 107644 | 997 | 0.90 | 0.82 | 0.98 |  |
| Q4 | 107651 | 952 | 0.89 | 0.81 | 0.97 |  |
| *P* for trend |  |  |  |  |  | .002 |
|  | Model 3 | | | | | |
| continuous, per IQR increase | 367065 | 3586 | 0.94 | 0.90 | 0.98 |  |
| Q1 | 91271 | 1000 | Ref. | Ref. | Ref. |  |
| Q2 | 91728 | 932 | 0.96 | 0.88 | 1.05 |  |
| Q3 | 91973 | 848 | 0.90 | 0.82 | 0.99 |  |
| Q4 | 92093 | 806 | 0.89 | 0.81 | 0.97 |  |
| *P* for trend |  |  |  |  |  | .005 |

Abbreviations: NAFLD, non-alcoholic fatty liver disease; HR, hazards ratio; CI, confidence interval; IQR, interquartile range; Ref, reference.

*P* value for trend calculated treating the telomere length concentrations (quartile) as a continuous variable.

Model 1: Unadjusted.

Model 2: Adjusted for age, gender, ethnicity, Townsend deprivation index, BMI, Cholesterol, hypertension, and diabetes.

Model 3: Model 2 + lifestyle and air pollution score.

Table S8 Sensitivity analysis: after excluding participants diagnosed with NAFLD within the first2-years of follow-up

| Variables | N | Case | HR | Lower 95%CI | Upper 95%CI |  |
| --- | --- | --- | --- | --- | --- | --- |
|  | Model 1 | | | | | |
| continuous, per IQR increase | 467573 | 4534 | 0.88 | 0.85 | 0.91 |  |
| Q1 | 116869 | 1271 | Ref. | Ref. | Ref. |  |
| Q2 | 116903 | 1180 | 0.92 | 0.85 | 0.99 |  |
| Q3 | 116902 | 1071 | 0.83 | 0.76 | 0.90 |  |
| Q4 | 116899 | 1012 | 0.78 | 0.72 | 0.84 |  |
| *P* for trend |  |  |  |  |  | <.0001 |
|  | Model 2 | | | | | |
| continuous, per IQR increase | 467573 | 4534 | 0.93 | 0.89 | 0.96 |  |
| Q1 | 116869 | 1271 | Ref. | Ref. | Ref. |  |
| Q2 | 116903 | 1180 | 0.95 | 0.88 | 1.03 |  |
| Q3 | 116902 | 1071 | 0.89 | 0.82 | 0.97 |  |
| Q4 | 116899 | 1012 | 0.87 | 0.80 | 0.95 |  |
| *P* for trend |  |  |  |  |  | .0004 |
|  | Model 3 | | | | | |
| continuous, per IQR increase | 467573 | 4534 | 0.93 | 0.89 | 0.97 |  |
| Q1 | 116869 | 1271 | Ref. | Ref. | Ref. |  |
| Q2 | 116903 | 1180 | 0.95 | 0.88 | 1.03 |  |
| Q3 | 116902 | 1071 | 0.89 | 0.82 | 0.97 |  |
| Q4 | 116899 | 1012 | 0.88 | 0.81 | 0.95 |  |
| *P* for trend |  |  |  |  |  | .0007 |

Abbreviations: NAFLD, non-alcoholic fatty liver disease; HR, hazards ratio; CI, confidence interval; IQR, interquartile range; Ref, reference.

*P* value for trend calculated treating the telomere length concentrations (quartile) as a continuous variable.

Model 1: Unadjusted.

Model 2: Adjusted for age, gender, ethnicity, Townsend deprivation index, BMI, Cholesterol, hypertension, and diabetes.

Model 3: Model 2 + lifestyle and air pollution score.

Table S9 Sensitivity analysis: further adjusted for education and income

| Variables | N | NAFLD cases /person years | HR | Lower 95%CI | Upper 95%CI |  |
| --- | --- | --- | --- | --- | --- | --- |
| continuous, per IQR increase | 467848 | 4809 / 5860127 | 0.93 | 0.90 | 0.97 |  |
| Q1 | 116954 | 1356 / 1448756 | Ref. | Ref. | Ref. |  |
| Q2 | 116975 | 1252 / 1464364 | 0.95 | 0.88 | 1.03 |  |
| Q3 | 116959 | 1128 / 1470434 | 0.89 | 0.83 | 0.97 |  |
| Q4 | 116960 | 1073 / 1476574 | 0.89 | 0.82 | 0.96 |  |
| *P* for trend |  |  |  |  |  | .001 |

Abbreviations: NAFLD, non-alcoholic fatty liver disease; HR, hazards ratio; CI, confidence interval; IQR, interquartile range; Ref, reference.

*P* value for trend calculated treating the telomere length concentrations (quartile) as a continuous variable.

Adjusted for age, gender, ethnicity, Townsend deprivation index, BMI, Cholesterol, hypertension, diabetes, lifestyle, air pollution score, education, and income.

Table S10 Sensitivity analysis: further adjusted for alcohol intake frequency

| variables | N | NAFLD cases /person years | HR | Lower 95%CI | Upper 95%CI |  |
| --- | --- | --- | --- | --- | --- | --- |
| continuous, per IQR increase | 467848 | 4809 / 5860127 | 0.93 | 0.89 | 0.96 |  |
| Q1 | 116954 | 1356 / 1448756 | Ref. | Ref. | Ref. |  |
| Q2 | 116975 | 1252 / 1464364 | 0.95 | 0.88 | 1.03 |  |
| Q3 | 116959 | 1128 / 1470434 | 0.89 | 0.82 | 0.96 |  |
| Q4 | 116960 | 1073 / 1476574 | 0.88 | 0.81 | 0.95 |  |
| *P* for trend |  |  |  |  |  | .0003 |

Abbreviations: NAFLD, non-alcoholic fatty liver disease; HR, hazards ratio; CI, confidence interval; IQR, interquartile range; Ref, reference.

*P* value for trend calculated treating the telomere length concentrations (quartile) as a continuous variable.

Adjusted for age, gender, ethnicity, Townsend deprivation index, BMI, Cholesterol, hypertension, diabetes, lifestyle, air pollution score, and alcohol intake frequency.

Table S11 Sensitivity analysis: further adjusted for history of cancer and vascular/heart problems

| variables | N | NAFLD cases /person years | HR | Lower 95%CI | Upper 95%CI |  |
| --- | --- | --- | --- | --- | --- | --- |
| continuous, per IQR increase | 467848 | 4809 / 5860127 | 0.93 | 0.90 | 0.97 |  |
| Q1 | 116954 | 1356 / 1448756 | Ref. | Ref. | Ref. |  |
| Q2 | 116975 | 1252 / 1464364 | 0.95 | 0.88 | 1.03 |  |
| Q3 | 116959 | 1128 / 1470434 | 0.89 | 0.82 | 0.96 |  |
| Q4 | 116960 | 1073 / 1476574 | 0.88 | 0.81 | 0.95 |  |
| *P* for trend |  |  |  |  |  | .0005 |

Abbreviations: NAFLD, non-alcoholic fatty liver disease; HR, hazards ratio; CI, confidence interval; IQR, interquartile range; Ref, reference.

Vascular/heart problems includes heart attack, angina, stroke, and high blood pressure.

*P* value for trend calculated treating the telomere length concentrations (quartile) as a continuous variable.

Adjusted for age, gender, ethnicity, Townsend deprivation index, BMI, Cholesterol, hypertension, diabetes, lifestyle, air pollution score, history of cancer, and vascular/heart problems.

Table S12 Sensitivity analysis: added data on primary care

| Variables | N | Case | HR | Lower 95%CI | Upper 95%CI |  |
| --- | --- | --- | --- | --- | --- | --- |
| continuous, per IQR increase | 467108 | 6542 | 0.93 | 0.90 | 0.96 |  |
| Q1 | 116754 | 1821 | ref. | ref. | ref. |  |
| Q2 | 116788 | 1686 | 0.94 | 0.88 | 1.01 |  |
| Q3 | 116778 | 1582 | 0.91 | 0.85 | 0.98 |  |
| Q4 | 116788 | 1453 | 0.86 | 0.81 | 0.93 |  |
| *P* for trend |  |  |  |  |  | <.0001 |

Abbreviations: NAFLD, non-alcoholic fatty liver disease; HR, hazards ratio; CI, confidence interval; IQR, interquartile range; Ref, reference.

*P* value for trend calculated treating the telomere length concentrations (quartile) as a continuous variable.

Adjusted for age, gender, ethnicity, Townsend deprivation index, BMI, Cholesterol, hypertension, diabetes, lifestyle, air pollution score.

Table S13 Associations of telomere length with PDFF and PDFF-defined NAFLD

|  | β (95%CI) | OR (95%CI) |
| --- | --- | --- |
| Model 1 |  |  |
| Continuous, per IQR increase | -0.25 (-0.31, -0.18) | 0.89 (0.87, 0.92) |
| Quartile 1 | Ref. | Ref. |
| Quartile 2 | -0.13 (-0.28, 0.01) | 0.94 (0.88, 1.00) |
| Quartile 3 | -0.27 (-0.41, -0.12) | 0.88 (0.82, 0.94) |
| Quartile 4 | -0.51 (-0.65, -0.36) | 0.79 (0.74, 0.85) |
| P for trend | <.0001 | <.0001 |
|  |  |  |
| Model 2 |  |  |
| Continuous, per IQR increase | -0.12 (-0.17, -0.06) | 0.95 (0.92, 0.98) |
| Quartile 1 | Ref. | Ref. |
| Quartile 2 | -0.03 (-0.16, 0.11) | 0.98 (0.91, 1.05) |
| Quartile 3 | -0.08 (-0.22, 0.05) | 0.95 (0.88, 1.02) |
| Quartile 4 | -0.21 (-0.34, -0.08) | 0.90 (0.84, 0.97) |
| P for trend | 0.001 | 0.005 |
|  |  |  |
| Model 3 |  |  |
| Continuous, per IQR increase | -0.11 (-0.17, -0.05) | 0.95 (0.92,0.98) |
| Quartile 1 | Ref. | Ref. |
| Quartile 2 | -0.02 (-0.16, 0.11) | 0.98 (0.91,1.05) |
| Quartile 3 | -0.08 (-0.22, 0.05) | 0.95 (0.88,1.02) |
| Quartile 4 | -0.2 (-0.33, -0.07) | 0.91 (0.84,0.98) |
| P for trend | 0.002 | 0.005 |

Abbreviations: PDFF, proton density fat fraction; OR, odds ratio; CI, confidence interval; IQR, interquartile range; Ref, reference.

*P* value for trend calculated treating the telomere length concentrations (quartile) as a continuous variable.

Model 1: Unadjusted.

Model 2: Adjusted for age, gender, ethnicity, Townsend deprivation index, BMI, cholesterol, hypertension, and diabetes.

Model 3: Model 2 + lifestyle and air pollution score.

Table S14 Additive and multiplicative interactions between telomere length and other risk factors on NAFLD incidence

| Variables | Low TL | | Intermediate TL | | *P* for interaction |
| --- | --- | --- | --- | --- | --- |
|  | RERI (95% CI) | AP (95% CI) | RERI (95% CI) | AP (95% CI) |  |
| Age |  |  |  |  | 0.40 |
| >60 years old | -0.02 (-0.18, 0.14) | -0.01 (-0.14, 0.12) | -0.02 (-0.17, 0.14) | -0.02 (-0.16, 0.12) |  |
| Air pollution score |  |  |  |  | 0.60 |
| Intermediate | 0.08 (-0.11, 0.27) | 0.06 (-0.10, 0.22) | -0.02 (-0.22, 0.17) | -0.02 (-0.21, 0.16) |  |
| High | 0.03 (-0.17, 0.22) | 0.02 (-0.13, 0.17) | -0.06 (-0.26, 0.13) | -0.05 (-0.22, 0.11) |  |
| Lifestyle index |  |  |  |  | 0.18 |
| Unfavorable | 0.08 (-0.09, 0.25) | 0.06 (-0.07, 0.18) | 0.1 (-0.06, 0.27) | 0.08 (-0.05, 0.21) |  |
| PRS |  |  |  |  | 0.18 |
| Intermediate | -0.1 (-0.33, 0.13) | -0.08 (-0.25, 0.09) | -0.16 (-0.39, 0.08) | -0.12 (-0.31, 0.06) |  |
| High | **0.29 (0.05, 0.54)** | **0.14 (0.02, 0.26)** | -0.1 (-0.35, 0.15) | -0.06 (-0.21, 0.09) |  |

Abbreviations: TL, telomere length; NAFLD, non-alcoholic fatty liver disease; RERI, relative excess risk due to interaction; AP, attributable proportion due to interaction; CI, confidence interval; PRS, polygenic risk score;

To estimate RERI and AP, the high TL category and < 60 years old, low APS, healthy lifestyle, low PRS were the reference categories, respectively.

Genetic analysis adjusted for age, gender, ethnicity, Townsend deprivation index, BMI, cholesterol, hypertension, diabetes, lifestyle, air pollution score, the top 10 genetic principal components, and genotyping batch.

Other analysis adjusted for age, gender, ethnicity, Townsend deprivation index, BMI, cholesterol, hypertension, diabetes, lifestyle and air pollution score.
